# Nutritional, Physicochemical and Structural Parameters of *Mauritia flexuosa* Fruits and By-Products for Biotechnological Exploration of Sustainable Goods

**DOI:** 10.17113/ftb.60.02.22.7106

**Published:** 2022-06

**Authors:** Joilane Alves Pereira-Freire, Jailane de Souza Aquino, Ana Regina Nascimento Campos, Vicente Galber Freitas Viana, Joaquim Soares da Costa Júnior, Jurandy do Nascimento Silva, Arkellau Kenned Silva Moura, Antônia Maria das Graças Lopes Citó, Regilda Saraiva dos Reis Moreira-Araújo, Karoline de Macêdo Gonçalves Frota, Stella Regina Arcanjo Medeiros, Paulo Michel Pinheiro Ferreira

**Affiliations:** 1 Department of Nutrition, Federal University of Piauí, Cícero Eduardo, 646000-000, Picos, Brazil; 2 Department of Nutrition, Federal University of Paraíba, Campus I, 58059-900, João Pessoa, Brazil; 3 Department of Chemistry Engineer, Federal University of Campina Grande, Aprígio Velosos, 882, 58429-900, Campina Grande, Brazil; 4 Federal Institute of Education, Science and Technology of Piauí, Pedro Freitas Avenue, 1020, 64018-000, Teresina, Brazil; 5 Laboratory of Experimental Cancerology (LabCancer), Department of Biophysics and Physiology, Federal University of Piauí, Universitária Avenue, 64049-550, Teresina, Brazil; 6 Laboratory of Organic Geochemistry, Department of Chemistry, Federal University of Piauí, Universitária Avenue, 64049-550, Teresina, Brazil; 7 Department of Nutrition, Federal University of Piauí, Universitária Avenue, 64049-550, Teresina, Brazil

**Keywords:** fatty acids, phytosterols, rheology, scanning electron microscopy, nutritional composition

## Abstract

**Research background:**

Commercialization of *Mauritia flexuosa* (buriti) fruits in Brazil is at an early stage. Herein, we evaluate the nutritional value of pulp, peel and endocarp samples from buriti fruits, perform macroscopic and microscopic evaluations and analyze their physicochemical properties.

**Experimental approach:**

Size and mass, pH, sugar and protein contents, soluble/insoluble fiber, total titratable acidity and energy value of the samples were analyzed. Mineral profiling was performed by energy dispersive X-ray fluorescence spectrometry, and fatty acids and phytosterols were determined by gas chromatography-mass spectrometry. Samples were also submitted to differential scanning calorimetry coupled to a thermal analyzer, and microstructure, morphology, surface and viscosity were evaluated by scanning electron microscopy (SEM) and X-ray diffraction (XRD) with copper radiation. Rheological behavior was also studied.

**Results and conclusions:**

Lyophilized pulp had higher nutritional content of minerals, proteins, carbohydrates and energy than *in natura* pulp. Lyophilized pulp and its by-products showed suitable yields (>17.31%) and low *a*_w_, and potassium, manganese and monounsaturated fatty acid contents. Peels showed elevated amounts of saturated and polyunsaturated fatty acids and phytosterols (β-sitosterol and stigmasterol), and endothermic behavior. The reductions of calcium, magnesium and manganese ranging from 18.5 to 22.7% were observed following the lyophilization. Drying processes generated semi-crystalline powders. Both peels and endocarp contained higher amounts of insoluble fiber and lower contents of sugars. Similar results were obtained by microscopic morphological analysis, differential scanning calorimetry and XRD analysis. Pulp and endocarp exhibited pseudoplastic non-Newtonian behavior, and flow behavior index values were lower than 1, while peels presented dilatant behaviour. Thus, physicochemical and nutritional characterization of pulp and by-products, such as peels and endocarp, are essential to support scientific research and exploration of new sustainable products.

**Novelty and scientific contribution:**

Processing and conservation techniques, like lyophilization, maintain the good quality of nutritional contents and bioactive compounds of buriti whole fruits, and can be used to extend their shelf life, preserve alimentary characteristics and provide wider purposes and availability. Such parameters may generate income and food security for local and regional communities.

## INTRODUCTION

*Mauritia flexuosa* L., popularly known as buriti, belongs to the Brazilian Amazon and Cerrado biomes (*1*). In producing regions, buriti fruit pulp or mesocarp is mainly used for the preparation of cookies (*2*), sweets, juices and ice cream, as well as for oil consumption (*3*).

Buriti pulp and oil have been investigated for their nutritional and sensory values and are even considered functional foods due to their content of bioactive compounds such as carotenoids, tocopherols, phenolic compounds and radical scavenging capacity (*4*-*6*). This nutritional value may contribute to the buriti pharmacological and medicinal properties, namely hypoglycaemic (*5*), antitumor (*7*), antioxidant, chemopreventive (*5*, *6*) and antimicrobial (*7*), and thus improve public health. Moreover, bioactive compounds found in buriti fruits have also aroused interest in the cosmetic, biofuel and nanotechnology industries (*3*, *5*).

However, there are no reliable estimations about the commercialization of buriti fruits and by-products in Brazil, since such production is still at an early stage, although these fruits present great biotechnological and economic potential (*2*-*4*). Buriti (*Mauritia flexuosa*), gueroba (*Syagrus oleracea* Becc.) and aricuri (*Scheelea phalerata* Mart. ex Spreng) are native, exotic fruits from Brazilian Arecaceae family with fragmented data about their phytochemical and ethnopharmacological aspects despite their wide popular use due to functional and nutraceutical properties (*5*, *8*). Using conservation methods such as lyophilization, it is possible to preserve bioactive compounds, and make products more convenient for consumption and storage, thus consequently, extend their shelf life and better availability for commercialization, which reduces the influence of seasonality on fruits (*9*). Additionally, physicochemical, rheological and technical analyses, such as energy dispersive X-ray spectroscopy, chromatography coupled with mass spectrometry, differential scanning calorimetry, X-ray diffraction and scanning electron microscopy in food matrices, help to determine nutritional composition and biotechnological potential to better target sustainable usage for manufacturing purposes (*10*). Thus, this study evaluates the nutritional value of pulp, peel and endocarp samples of buriti (*Mauritia flexuosa*) fruits, using macroscopic and microscopic evaluations and analyzes their physicochemical properties.

## MATERIALS AND METHODS

### Collection of samples

A sample of *Mauritia flexuosa* (buriti) was deposited in the Graziela Barroso Herbarium at Federal University of Piauí (UFPI) (voucher specimen 30567). The sample (#A690444) was registered in SisGen (Sistema Nacional de Gestão do Patrimônio Genético e do Conhecimento Tradicional Associado, *i.e.* National System of Management of Genetic Heritage and Associated Traditional Knowledge, Glück Informatica, Rio de Janeiro, Brazil) (*11*) according to the Brazilian biodiversity legislation (Federal Law No. 13.123/2015) (*12*). Approximately 300 fruits were collected in Água Branca, Piauí, Brazil, in December 2014 (latitude: 5°54’50.5” S; longitude: 42°38’03.4” W) and taken to the Federal Institute of Education, Science and Technology of Piauí, Teresina, Brazil. Fruit selection, cleaning, separation, lyophilization, packaging, refrigeration and pulverization were carried out according to Pereira-Freire *et al*. (*6*). Briefly, all fruits were separated into pulp, peel and endocarp and frozen at −70 °C. For the lyophilization, fruits were placed on a stainless-steel tray of lyophilizer model L101 (Liotop, São Carlos, Brazil). Lyophilization conditions (temperature: 40 °C, vacuum pressure: <500 mmHg, lyophilization rate: 1m/h) were controlled during 72 h. Afterwards, the fruits were packaged in plastic bags under refrigeration at 4 °C before processing into powder using a rotor mill (0.08 mm; Retsch, Haan, Germany).

### Macroscopic characterization and yield of fruits

The yield before lyophilization (*Y*_1_) of samples was obtained on the total fruit mass basis. The average mass (g) of fruits was measured on a semi-analytical scale, and longitudinal and transversal diameter was determined using a digital caliper with 0−150 mm capacity and resolution of 0.01 mm (Digmess, São Paulo, Brazil). Sampling for physical measurements: average fruit mass, transverse diameter (cm) and longitudinal diameter (cm) was performed by using 10% of samples, *i.e.* 30 units of fresh fruits (1.46 kg). All measurements were done in triplicate.

To calculate the yield of all samples after lyophilization (*Y*_2_), the following equation was used:

*Y*_2_=(*Y*_i_–*Y*_f_)/100 /1/

where *Y*_i_ is the yield of samples before lyophilization (*in natura*) and *Y*_f_ is the yield of samples after lyophilization.

### Physicochemical characterization

To measure pH, a bench potentiometer (model EEQ9002G-2; A. Científica, Santo André, Brazil) was used. Sugar content in °Brix was measured using a refractometer (DR500; New Instruments, Piracicaba, Brazil), total titratable acidity was determined by the volumetric method, water activity (*a*_w_) (Novasina, Aqualab, São José dos Campos, Brazil) and moisture content were determined by drying in an oven at 105 °C to constant mass, ash content was measured by incineration in a muffle oven at 550 °C, protein content was measured by the Kjeldahl method, lipid content was measured by direct hexane-based extraction using the Soxhlet technique, and carbohydrates were calculated by difference method (*13*). The total dietary fiber, soluble and insoluble contents were quantified by the enzymatic-gravimetric method (*14*). All analyses were performed in triplicate.

The total energy value (*E*) was calculated based on the conversion factors for protein (4 kcal/g or 16 736 kJ), lipids (9 kcal/g or 37 156 kJ) and carbohydrates (4 kcal/g or 16 736 kJ), expressed in kilocalories per 100 g of dried sample (*15*), using the following equation:

*E*=(*E*(protein)·4)+(*E*(lipid)·9)+(*E*(carbohydrate)·4) /2/

### Mineral composition

The mineral composition of pulp, peel and endocarp was determined by energy dispersive X-ray fluorescence spectrometry using energy dispersive X-ray spectroscopy (EDX-720; Shimadzu, Kyoto, Japan). Parts were placed in their own sample support separately, sealed with thin polypropylene film to avoid extrusion of samples by activating the vacuum and analyzed (*16*).

#### Identification of fatty acids and phytosterols by gas chromatography-mass spectrometry

The fatty acid profile was determined after the esterification of lipid extracts to obtain methyl esters (*17*), and fatty acids and phytosterols were quantified using GC-17A gas chromatograph with QP5050A mass spectrometer (GC-MS) (Shimadzu). A total of 5 mL of methanolic sodium hydroxide solution (5%) was added to the oil, and the mixture was refluxed for 5 min. Then, 10 mL of esterifying reagent (2 g NH_4_Cl in 60 mL MeOH refluxed in concentrated sulfuric acid) were added and refluxed for an additional 5 min. The obtained mixture was transferred to a separatory funnel, and 20 mL of distilled water and 30 mL of ethyl ether were added. The ethereal phase was separated, dried using anhydrous sodium sulfate, filtered and evaporated on a rotary evaporator at 30 °C (*17*).

Chromatographic separation was performed by a capillary chromatographic column Rxi-5HT (5% diphenyl and 95% dimethylpolysiloxane) (Restek, Bellefonte, PA, USA), 30 m×0.25 mm×0.25 µm, with the following temperatures: initial temperature 70 °C (kept for 2 min) followed by a heating ramp of 6 °C/min to a final temperature 310 °C for 10 min. The quadrupole-type mass spectrometer was operated in scan mode in the mass range of 47−600 Da. The ion source was set to operate in electron ionization mode at 70 eV. The total scan time for the chromatographic run was 52 min, including a 3-minute solvent delay. Fatty acids and phytosterols were identified by comparing the fragmentation pattern and retention times observed in the mass spectra with library software (*18*). The results are expressed as the percentage of the area of each signal of the total fatty acid signal area. Therefore, quantification was not performed since percentages of each constituent were calculated from the integration of the area, and relative but not absolute values of the constituents in each sample were determined.

#### Differential scanning calorimetry

Differential scanning calorimetry (DSC) curves were obtained in a differential scanning calorimetric module DSC 910 (TA Instruments, Waters, New Castle, DE, USA) (heat flow type) coupled to a TA2000 (TA Instruments, Waters) thermal analyzer, using aluminum sample support under air and nitrogen atmosphere. To demonstrate the influence of the parameters, 3 mg of heated sample were used under a dynamic atmosphere of synthetic air (100 mL/min) and increasing heating (2.5, 5, 10, 15, 20 and 40 °C/min) for each sample (*19*).

#### X-ray diffraction

Triturated samples were fixed in a glass holder, and readings were carried out in an X-ray diffractometer (model MiniFlex; Applied Rigaku Technologies, Inc., Austin, TX, USA) with copper radiation (Cu Kα=1.5418 Å) operating at 40 kV and 25 mÅ equipped with a curved pyrolytic graphite monochromator positioned between the sample and the scintillation detector. X-ray diffractograms were obtained with angle 2*θ* ranging from 3 to 120° at a step time 2°/min (*20*).

#### Rheological analysis

The rheological properties of the pulp, peel and endocarp were determined using Searle principle in a concentric cylindrical roller (rheometer model R/S plus SST 2000; Brookfield, Stoughton, MA, USA) at 25 °C. The equipment provided shear stress and strain rate data through Rheo3000 v. 2.2.28 software (Brookfield AMETEK) (*21*).

Rheological analyses were obtained by deformation rate ranging from 0 to 500/s (upward curve) and from 500 to 0/s (downward curve), with a time of 1 min and reading of 25 points for each curve. Curves of apparent viscosity as a function of the strain rates were plotted using the experimental viscosity data and theoretical values calculated from the ideal model (Ostwald-de-Waele), taking the following into consideration:

*τ*=*K*·*γ*^n^ /3/

where *τ* is shear stress (Pa), *K* is consistency index (Pa·s), *n* is behavior index (dimensionless) and *γ* is deformation rate (s^-1^) (*22*, *23*).

### Structural characteristics by scanning electron microscopy

Microstructure, morphology and surface evaluations of pulp, peel and endocarp powders were performed by scanning electron microscopy (SEM, SSX-550 Superscan; Shimadzu). The powders were fixed on metal support under vacuum and metallized with a thin gold film. Micrographs were obtained at different magnifications using acceleration voltages of 8 to 15 kV. The metal plate was covered with platinum (model K 550 metallizer; Emitech, Ashford, UK) and operated at 10 kV, providing a coverage of approx. 25 μm. Images were captured and scanned.

### Statistical analysis

All analyses were performed in triplicate, and the results were analyzed by ANOVA followed by Tukey’s test considering a 5% significance level (p<0.05) using SPSS software v. 24.0 for Windows (*24*). All analyses were performed in triplicate.

## RESULTS AND DISCUSSION

### Physical characteristics of fresh and lyophilized buriti fruits

In general, most buriti fruits had the typical ellipsoid-oblong shape, in a very similar way to the globular-oblong shape reported by Milanez *et al*. (*1*) and Lorenzi *et al*. (*8*). The coefficients of variation showed low uniformity of fruits in relation to the analyzed parameters (Table 1) given that cultivation is not planned, as occurs in other countries of South America. Seed and pulp mass, and consequently, yield (34.04 and 22.6%, respectively) were lower than those described by Milanez *et al*. (*1*). These results indicate that the production of fruits is strongly influenced by environmental factors such as temperature, solar radiation and rainfall (*1*). Consequently, such factors alter the yield of fresh and processed fruits.

**Table 1:** Physical characteristics and yield of buriti (*Mauritia flexuosa*) fruits before and after lyophilization

Parameter	Mean±S.D.	CV/%	*Y*_1_/%	*Y*_2_/%
*m*(fruit)_total_/g*	48.7±1.3	2.7	nd	nd
*m*(pulp)/g	11.0±0.9	7.9	22.6	17.3
*m*(peel)/g	13.0±0.6	4.5	26.7	26.0
*m*(endocarp)/g	8.1±0.8	9.7	16.6	20.7
*m*(seed)/g	16.6±0.5	2.9	34.0	n.d.
*d*_transverse_/cm	4.3±0.2	5.0	n.d.	n.d.
*d*_longitudinal_/cm	5.3±0.5	9.1	n.d.	n.d.

S.D.=standard deviation. n.d.=not detected. CV=coefficient of variation, *Y*_1_ and *Y*_2_=yield of samples before (*in natura*) and after lyophilization, *d*=diameter. *N(fruit)=30 sampled out of 300 fruits

After lyophilization, peels (26.0%) and endocarp (20.72%) had higher yields (Table 1) and lower *a*_w_ (0.25 and 0.38; Table 2) than pulp (p<0.05). It is noteworthy that pulp yield is a very important quality parameter for the production of dehydrated products containing 15-20% moisture, caramels, honey and candies, since fruits with high pulp yield have a higher yield of product after processing, a clear criterion of productivity. Moreover, peels and endocarp have too low *a*_w_ values for microbial growth/proliferation, an excellent feature for future uses in food industries and fabrication of cookies, crackers, breakfast cereals, dry pet food, peanut butter, whole milk powder, dried vegetables, corn starch and potato chips (*25*).

**Table 2:** Physicochemical characteristics of pulp and by-products of buriti (*Mauritia flexuosa*) fruits

Parameter	Fresh pulp	Lyophilized pulp	Lyophilized peel	Lyophilized endocarp
*a*_w_	(0.96±0.00)^a^	(0.65±0.00)^b^	(0.25±0.00)^c^	(0.38±0.00)^d^
pH	(3.94±0.03)^b^	(4.08±0.02)^a^	(3.52±0.02)^d^	(3.71±0.02)^c^
*w*(TTA)/%	(7.60±0.23)^b^	(8.1±0.4)^a^	(2.61±0.09)^d^	(4.1±0.1)^c^
*w*(TSS)/%	(7.73±0.06)ª	(5.77±0.35)^b^	(2.8±0.2)^c^	(1.3±0.2)^d^
*w*(moisture)/%	(54.8±0.6)^a^	(4.8±0.2)^b^	(3.3±0.2)^c^	(5.21±0.08)^d^
*w*(mineral)/(mg/100 g)*	(2.27±0.05)^c^	(2.18±0.02)^d^	(2.9±0.2)^b^	(4.64±0.03)ª
*w*(protein)/%*	(2.47±0.07)^d^	(5.6±0.2)^a^	(4.13±0.02)^b^	(4.8±0.2)^c^
*w*(carbohydrate)/(mg/100 g)*	(15.1±0.3)^d^	(35.7±0.5)^c^	(77.5±0.2)ª	(73.8±0.1)^b^
*w*(lipid)/%*	(26.6±0.3)^b^	(51.67±0.09)^a^	(12.13±0.02)^c^	(11.54±0.08)^d^
*w*(total fiber)/%*	(38.0±0.3)^b^	(38.9±0.6)^b^	(50.5±0.6)^a^	(28.14±0.05)^c^
*w*(IF)/%	(27.3±0.4)^b^	(28.8±1.0)^b^	(50±1)^a^	(24.66±0.01)^c^
*w*(SF)/%	(10.6±0.3)^a^	(10.1±0.2)^a^	(0.55±0.03)^c^	(3.48±0.08)^b^
*w*(IF)/*w*(SF)	(2.6±0.4)^a^	(2.8±0.5)^a^	(90.8±0.7)^a^	(7.09±0.04)^a^
*E*/(kcal/100 g)	(310±4)^d^	(630.3±0.7)^a^	(435.8±0.9)^b^	(418.3±0.7)^c^

Values are expressed as mean±S.D. Different letters in same row differ according to Tukey’s test (p<0.05). TTA=total titratable acidity, TSS=total soluble solids, *on wet mass basis, IF and SF=insoluble and soluble fiber

### Physicochemical properties of buriti fruit samples

Lyophilized pulp had higher nutritional contents of ash (*i.e.* minerals) (2.18±0.02) mg/100 g, protein (5.6±0.2) %, carbohydrates (35.7±0.5) mg/100 g, lipids (51.67±0.09) % and energy (630.3±0.7) kcal/100 g than *in natura* pulp ((2.27±0.05) mg/100 g, (2.47±0.07) %, (15.1±0.3) mg/100 g, (26.6±0.3) % and (310±4) kcal/100 g, respectively, p<0.05). This is obviously related to the reduction in water content after lyophilization. Similarly, lyophilized peels and endocarp were richer in minerals, protein, carbohydrates and energy, but poorer in lipids (p<0.05) (Table 2). These outcomes indicate that pulp is a good source of lipids. Indeed, buriti pulp oil has carotenoids, tocopherols and monounsaturated fatty acids, which makes it a valuable product with functional potentialities (*4*-*6*).

Previous investigations have demonstrated that buriti fruits are sources of carbohydrates, whose amount is controlled by the levels of biosynthetic enzymes and gene expression (*26*). In a similar way, lyophilized peel samples had higher fiber mass fraction ((50.5±0.6) %), higher content of insoluble ((50±1) %) and lower values of soluble fiber ((0.55±0.03) %) than fresh pulp samples ((38.0±0.3), (27.3±0.4) and (10.6±0.3) %, respectively, p<0.05). This can be considered an advantage since insoluble fiber is widely used to increase the content of compounds added to foods to improve the rheological properties of dietary products, increasing satiety, and the volume and faecal mass, which clearly improves digestive system performance (*27*).

Buriti pulp, peels and endocarp had lower contents of soluble fiber than the by-products from other fruits such as mango (28.2%), passion fruit (35.5%) and guava (11.1%) (*28*). However, lyophilized buriti peel had a greater quantity of insoluble fiber than mango (41.5%) and passion fruit (46%) by-products (*28*). Soluble fiber is advantageous because when incorporated into food, it increases viscosity and the ability to form gels and/or act as emulsifier. In addition, consumption of soluble fiber-rich foods may reduce blood glucose and cholesterol levels (*29*). In this context, 30 g of pulp, peel or endocarp samples can be used to provide more than 15% of the dietary reference intake (DRI) (*30*), which recommends consumption of 21 to 38 g/day dietary fiber, taking into consideration different human factors, such as age, physiological condition and sex.

It is worth noting that the consumption of fresh buriti fruits has limitations, mainly due to high water loss and susceptibility to chilling injuries when stored under refrigeration (*31*). Therefore, processing buriti fruits, such as by lyophilization, and its use for the development of new comestible products is effective for nutrient conservation, increased production, shelf-life extension and availability independent of season (*32*). Therefore, buriti pulp used for processing should derive from by-products if their chemical and physicochemical characterization is acceptable, which supports a sustainable destination and nutritional applications.

### Mineral profile of buriti fruit samples

Lyophilized pulp had higher mass fraction of potassium (712.0±0.4) and chlorine (72.0±0.2) mg/100 g, peels had more phosphorus (26.0±0.1) and iron (19.00±0.02) mg/100 g, and copper (1.00±0.01) µg/100 g, and endocarp was richer in potassium (713.0±0.3) and calcium (159.0±0.1) mg/100 g, and copper (1.00±0.01) µg/100 g than fresh pulp (Table 3, p<0.05). Interestingly, chromium ((4.00±0.01) µg/100 g) was detected only in lyophilized peels. In general, the reductions of calcium, magnesium and manganese ranging from 18.5 to 22.7% were observed following the lyophilization.

**Table 3:** Mineral composition of pulp and by-products of buriti (*Mauritia flexuosa*) fruits

Mineral	Fresh pulp	Lyophilized pulp	Lyophilized peel	Lyophilized endocarp
*w*(mineral)/(mg/100 g)			
Potassium	(672.0±0.4)^b^	(712.0±0.4)^a^	(595.0±0.3)^c^	(713.0±0.3)^a^
Calcium	(148.0±0.3)^b^	(120.0±0.3)^c^	(101.0±0.4)^d^	(159.0±0.1)^a^
Chlorine	(65.0±0.2)^b^	(72.0±0.2)^a^	(53.0±0.1)^c^	(41.0±0.3)^d^
Magnesium	(50.0±0.3)^a^	(40.0±0.2)^b^	(41.0±0.1)^b^	(39.0±0.3)^b^
Phosphorus	(21.0±0.2)^b^	(19.0±0.1)^b^	(26.0±0.1)^a^	(11.0±0.1)^c^
Manganese	(18.0±0.2)^a^	(14.0±0.2)^b^	(14.0±0.1)^b^	(18.0±0.1)^a^
Sulfur	(12.0±0.2)^a^	(12.0±0.2)^a^	(11.0±0.1)^a^	(6.0±0.3)^b^
Iron	(2.00±0.02)^b^	(2.00±0.04)^b^	(19.00±0.02)^a^	(2.00±0.03)^b^
Zinc	(1.00±0.01)^b^	(1.00±0.02)^b^	(1.00±0.02)^a^	(1.00±0.02)^b^
*w*(mineral)/(µg/100 g)				
Copper	(0.40±0.01)^c^	(0.40±0.01)^c^	(1.00±0.01)^b^	(1.00±0.01)^a^
Chromium	n.d.	n.d.	(4.00±0.01)^a^	n.d.

Values are expressed as mean±S.D. Different letters in the same row differ according to Tukey’s test (p<0.05). n.d.=not detected

Lyophilization preserves the unique properties of bioproducts such as minerals, vitamins, bioactive compounds, color and flavor (*9*), although Marques *et al*. (*32*) reported a reduction in mineral content after lyophilization and rehydration of freeze-dried fruits. Peels reveal higher contents of potassium, iron and manganese and lower contents of copper and zinc than Amazonian native fruits, such as biribá (*Rollinia mucosa*), cubiu (*Solanum sessiliflorum* Dunal), sapota (*Quararibea cordata* H.B.K.) and umari (*Poraqueiba sericea* Tulasne) (*33*).

All analyzed buriti fruit samples are considered suitable sources of potassium and manganese (*30*, *34*). Potassium is essential for blood pressure control and improves cardiovascular function (*35*), and manganese is considered a structural co-enzymatic component and protects cell membranes against oxidative processes (*36*).

### Fatty acid and phytosterol profiles

Fresh or lyophilized pulp had a higher mass fraction of total monounsaturated fatty acids (both 80.11%), especially oleic acid (79.15 and 80.11%, respectively; Table 4). Meanwhile, lyophilized peels had elevated mass fractions of total saturated (23.18%) and polyunsaturated (2.11%) fatty acids, and they were the only by-product containing phytosterols (β-sitosterol 1.37 and stigmasterol 0.37%). Such results are similar to those in the literature, since previous investigations report oleic (75.7%), palmitic (18.9%), linoleic (2.1%), arachidonic (1.7%), palmitoleic (0.3%) and stearic (1.3%) acids in fruits (*4*, *6*).

**Table 4:** Mass fractions of fatty acids and phytosterols and rheological parameters of pulp and by- products of buriti (*Mauritia flexuosa*) fruits

Parameter	Fresh pulp	Lyophilized pulp	Lyophilized peel	Lyophilized endocarp
*w*(fatty acid)/%			
Palmitic acid (C16:0)	15.96	17.71	20.81	16.72
Stearic acid (C18:0)	4.60	1.59	2.37	1.46
Total saturated fatty acids	20.56	19.30	23.18	18.18
Palmitoleic acid (C16:1)	n.d.	n.d.	0.77	n.d.
Oleic acid (C18:1)	79.15	80.11	67.39	78.28
Total monounsaturated fatty acids	79.15	80.11	68.16	78.28
Linoleic acid (C18:2)	n.d.	n.d.	2.11	1.71
Linolenic acid (C18:3)	n.d.	n.d.	n.d.	n.d.
Total polyunsaturated fatty acids	n.d.	n.d.	2.11	1.71
		*w*(phytosterol)/%		
β-sitosterol	n.d.	n.d.	1.37	n.d.
Stigmasterol	n.d.	n.d.	0.37	n.d.
Total phytosterols	n.d.	n.d.	1.74	n.d.
Total fatty acids and phytosterols	99.71	99.41	95.19	98.17
Rheological parameters				
*K*	n.d.	0.001±0.000	3.0±0.6	2.1±0.5
*n*	n.d.	1.47±0.07	0.38±0.04	0.30±0.04
R^2^	n.d.	0.99	0.86	0.89

n.d.=not determined, *K*=consistency index, *n*=flow behavior index

The identification and quantification of fatty acids in food is necessary given that clinical and epidemiological studies have established that the quantity and type of lipids have a great influence on cardiovascular risk factors and inflammatory processes (*37*). Essential long-chain fatty acids belonging to the family omega 6 and 3 have healthy effects on physiological processes, including the prevention and treatment of cardiovascular diseases, atherosclerosis, hypertriglyceridaemia, hypertension, cancer diabetes, arthritis and inflammation-related conditions (*37*, *38*). Indeed, we have recently reported the anti-edematogenic effect of epicarp and mesocarp aqueous extracts from buriti fruits against phlogistic agents (carrageenan, compound 48/80, histamine, serotonin and prostaglandin E_2_) and reduction of tissue inflammation and the migration of peritoneal leukocytes and TNF-α in mesocarp-treated mice, but only epicarp reduced inflammatory abdominal pain induced by acetic acid (*39*).

For the first time, this study has described the presence and quantity of phytosterols in lyophilized peels. Phytosterols reduce cholesterol absorption in the intestine by up to 30% by competition due to the structural similarity between molecules and decrease serum LDL cholesterol levels by 8-10% when 1.6-2.0 g of phytosterols are consumed daily (*40*). Although our study did not find phytosterols in the pulp samples, it should be pointed out that 100 g of buriti pulp often contains brassicasterol, campesterol, stigmasterol, β-sitosterol and sitostanol. Obviously, samples from Brazilian savannas have nutritional profile distinctions when compared with those from the Amazon region, explained, at least in part, by differences in the Amazon biome conditions (*5*).

### Thermoanalytical data

In recent decades, thermoanalytical techniques have received increasing attention in most areas of basic and applied chemistry. For natural products, it is worth mentioning that evaluation of their quality depends not only on the chemical composition of the product but also on the quality of the raw material and it reflects processing and storage conditions (*41*). Therefore, differential scanning calorimetry (DSC) is an additional analysis widely used to detect the thermotropic behavior of complex inorganic and organic materials, such as buriti samples, and variations in melting, boiling and sublimation points or disappearance of these records (*42*).

**Fig 1:**
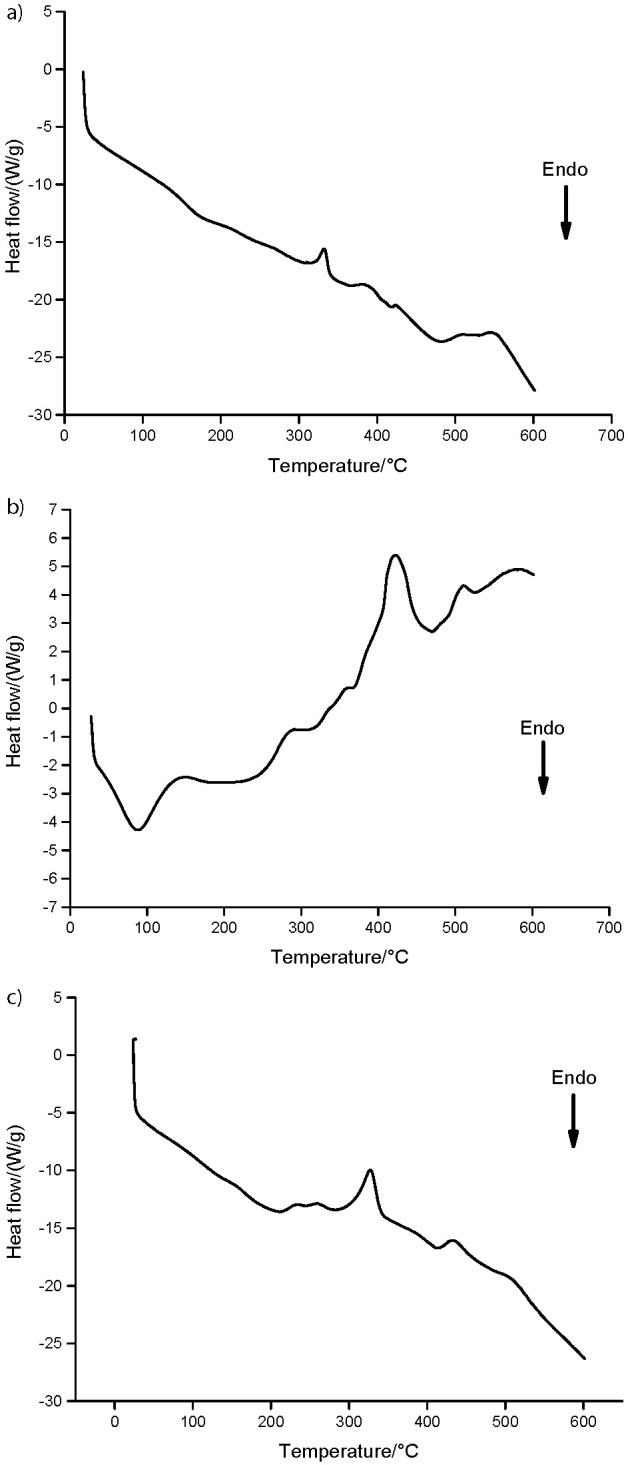
Differential scanning calorimetry (DSC) of: a) pulp, b) peel and c) endocarp samples of *Mauritia flexuosa* fruits

Fig. 1 illustrates DSC thermograms of pulp, peel and endocarp powders. The endothermic peak observed at 87.8 °C for peels refers to an endothermic reaction, *i.e.* melting and loss of residual water. Crystallization after glass transition and subsequent melting were observed, indicating that the sample was in an amorphous state with little crystallization by quenching after heating. Endothermic peaks were not observed in the pulp and endocarp samples, which may indicate that some components present in the peel have a higher affinity for water molecules. Therefore, higher temperatures are necessary for such elimination (which does not occur in lyophilization), or the material may have absorbed some moisture after drying. In addition, three exothermic peaks were observed between 240 and 530 °C, probably due to the degradation of some components in the peel, whose peak at 296 °C was found in thermograms of peel powder only (Fig. 1b).

The DSC curves for pulp and endocarp were similar (Figs. 1a and 1c), where exothermic degradation peaks were observed at 200 °C. A peak temperature of 327 °C found in endocarp samples was displaced to 331 °C in pulp, requiring a higher temperature to start the process. Moreover, it was also observed that for degradation of the pulp less energy was required than for the endocarp. This lower temperature necessary to disorganize the components of freeze-dried pulp suggests that physicochemical differences, such as the shape and distribution of fiber and the presence of lipids, have a pronounced influence on the thermal behavior (*42*).

The enthalpy (Δ*H*) was also measured by DSC analysis to determine qualitative parameters based on shape, position and number of peaks during heating or cooling, while the area under the curve supports quantitative examinations to recognize how external factors can affect DSC results (*43*).

### X-ray diffraction and microscopy observations

**Fig. 2:**
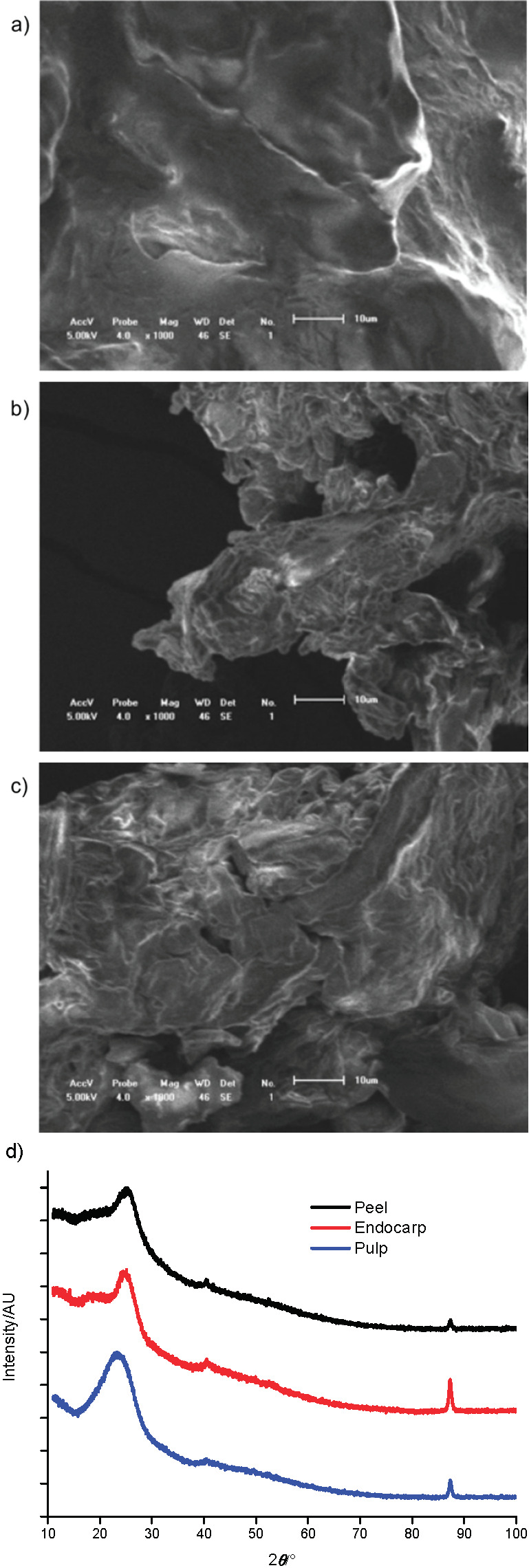
Morphological aspects of: a) pulp, b) peel and c) endocarp samples of buriti (*Mauritia flexuosa*) fruits analyzed by scanning electron microscopy at magnification of 1000×; d) X-ray diffractograms were obtained with 2*θ* from 3° to 120° at a step time 2°/min

Fig. 2 shows that drying processes generated semi-crystalline powders, characterized by the presence of larger and more intense peaks ranging from 15 to 25° diffraction angles (2*Ɵ*) and a lower intensity peak close to 90°. These results differ from those of other lyophilized fruits (*44*) and imply that constituents from buriti samples (minerals, proteins, lipids and crude fiber) alter the crystallinity of granules (*45*).

To confirm the XRD results, SEM of all samples was performed. The results corroborated XRD studies, since amorphous structures were visualized (Fig. 2) but with a semi-crystalline appearance.

Amorphous structures can be visualized in systems consisting of sugars. Buriti fruits are rich in different types of carbohydrates, and such structures are characterized by a non-crystalline structure in which there is no repetition of geometric unit cell or the presence of well-established flat faces, a finding more common in freeze-dried products (*46*). Therefore, the presence of sugars such as fructose, mainly in the pulp, as well as the use of lyophilization, are predisposing factors to generate amorphous microstructures. In the freeze-drying process, the glass transition temperature (*T*_g_) is exceeded during freezing, which makes the concentrated amorphous solution less viscous. Subsequently, product collapse may occur when ice is sublimated (*47*).

As described above, peels and endocarp contained higher mass fractions of insoluble components (fiber) than pulp and lower mass fractions of sugars. This composition was confirmed by comparable microscopic morphological analysis and similar thermograms and XRD curves.

### Rheological characteristics of buriti fruit samples

**Fig. 3:**
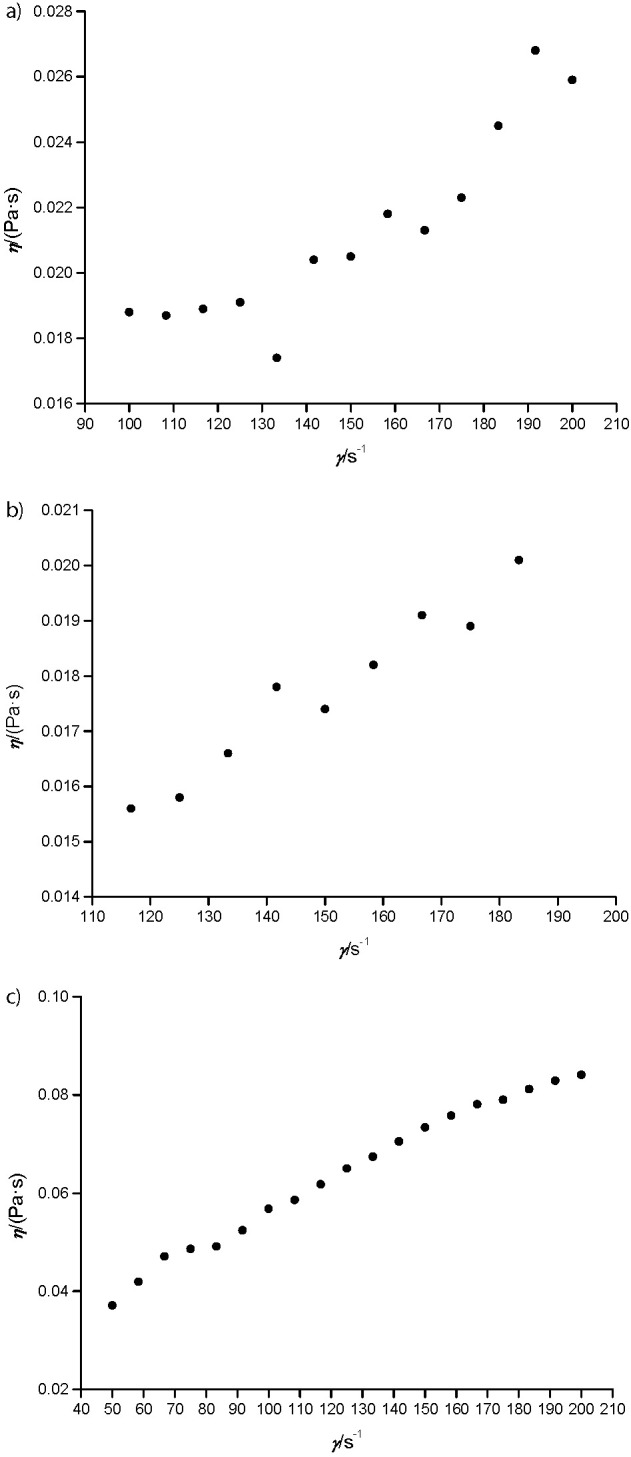
Rheological behavior of: a) pulp, b) peel and c) endocarp samples of *Mauritia flexuosa* fruits determined using a Searle principle in a concentric cylindrical roller. *η*=vicosity, *γ*=deformation rate

Pulp and endocarp showed pseudoplastic non-Newtonian behavior (Fig. 3), and the flow behavior index values were lower than 1 (*n*<1) at 25 °C. However, peel had dilatant behavior (*n*>1), similar to a suspension of insoluble particles (*48*), probably due to the insoluble/soluble fiber ratio that was about 90-fold higher (Table 2). Therefore, peels showed an inverse behavior since apparent viscosity increased proportionally to the deformation rate *γ*, which is related to the total dissolution of particles in the sample and direct contact among them, increasing viscosity (*49*).

## CONCLUSIONS

Lyophilized buriti fruit samples (pulp, peel and endocarp) contained high mass fractions of carbohydrates, lipids, fiber, potassium, manganese and monounsaturated fatty acids, and had suitable yield and low *a*_w_. The peels had increased mass fractions of saturated and polyunsaturated fatty acids, endothermic features and phytosterols. Drying resulted in semi-crystalline powders, whose amorphous properties were confirmed by microscopic examination. Peels and endocarp contained higher mass fraction of insoluble components (fiber) than the pulp and lower mass fraction of sugars. This composition was confirmed by comparable microscopic morphological analysis, and similar thermograms and XRD results. From a rheological point of view, pulp and endocarp showed pseudoplastic non-Newtonian behavior, while peels had dilatant behavior. Considering these aspects, physicochemical and nutritional characterization of pulp and by-products, such as peels and endocarp, are essential to support scientific research and exploration of new sustainable products. Therefore, processing and conservation techniques, such as lyophilization of whole buriti fruits, maintain the good quality of nutritional components and bioactive compounds, and they can be used to extend fruit shelf life, preserve alimentary characteristics and extend its usage and availability. Such molecular characteristics and properties may provide income and improve food safety in local and regional communities.

## References

[1] Milanez JT Neves LC Silva PMC Bastos VJ Shahab M Colombo RC Pre-harvest studies of buriti (*Mauritia flexuosa* L.F.), a Brazilian native fruit, for the characterization of ideal harvest point and ripening stages. Sci Hortic (Amsterdam). 2016;202:77–82. 10.1016/j.scienta.2016.02.026

[2] Aquino JS Vasconcelos MH Pessoa DCND Barbosa JK Prado JPS Magnani M Intake of cookies made with buriti oil (*Mauritia flexuosa*) improves vitamin A status and lipid profiles in young rats. Food Funct. 2016;7(10):4442–50. 10.1039/C6FO00770H 27713990

[3] Silva MF Lopes PS Silva CF Yoshida CMP. Active packaging material based on buriti oil – *Mauritia flexuosa* L. f. (*Arecaceae*) incorporated into chitosan films. J Appl Polym Sci. 2016;133(12):43210. 10.1002/app.43210

[4] Aquino JS Soares JKB Magnani M Stamford TCM Mascarenhas RJ Tavares RL Effects of dietary Brazilian palm oil (*Mauritia flexuosa* L.) on cholesterol profile and vitamin A and E status of rats. Molecules. 2015;20(5):9054–70. 10.3390/molecules20059054 25996211 PMC6272516

[5] Pereira-Freire JA Barros KBNT Lima LKF Martins JM Araújo YC Oliveira GLS Phytochemistry profile, nutritional properties and pharmacological activities of *Mauritia flexuosa.* J Food Sci. 2016;81(11):R2611–22. 10.1111/1750-3841.13529 30240016

[6] Pereira-Freire JA Oliveira GLDS Lima LKF Ramos CLS Arcanjo-Medeiros SR Lima ACS *In vitro* and *ex vivo* chemopreventive action of *Mauritia flexuosa* products. Evid Based Complement Alternat Med. 2018;2018:2051279. 10.1155/2018/2051279 29967646 PMC6008795

[7] Siqueira EP Andrade AA Souza-Fagundes EM Ramos JP Kohlhoff M Nunes YRF *In vitro* antibacterial action on methicillin-susceptible (MSSA) and methicillin-resistant (MRSA) *Staphylococcus aureus* and antitumor potential of *Mauritia flexuosa* L. f. J Med Plants Res. 2014;8(48):1408–17. 10.5897/JMPR2014.5688

[8] Lorenzi H, Noblick LR, Kahn F, Ferreira E, editors. Brazilian flora: Arecaceae (Palms). Nova Odessa, Brazil: Instituto Plantarum de Estudos da Flora; 2010.

[9] Nemzer B Vargas L Xia X Sintara M Feng H. Phytochemical and physical properties of blueberries, tart cherries, strawberries, and cranberries as affected by different drying methods. Food Chem. 2018;262:242–50. 10.1016/j.foodchem.2018.04.047 29751916

[10] Wahl V Khinast J Paudel A. Lyophilized protein powders: A review of analytical tools for root cause analysis of lot-to-lot variability. Trends Analyt Chem. 2016;82:468–91. 10.1016/j.trac.2016.05.012

[11] SisGen. National system of management of genetic heritage and associated traditional knowledge. Rio de Janeiro, Brazil: Government of Brazil; 2020. Available from: https://www.gov.br/pt-br/servicos/cadastrar-acesso-ao-patrimonio-genetico-e-ou-conhecimento-tradicional-associado (in Portuguese).

[12] Law no 13.123 of 20 May 2015. Brasilia, Brazil: Presidency of the Republic, General Secretariat for Legal Affairs; 2015. Available from: http://www.planalto.gov.br/ccivil_03/_ato2015-2018/2015/lei/l13123.htm.

[13] Official Method AOAC. 920.85/87-1920. Fat (crude) or ether extract in flour. Rockville, MD, USA: AOAC International; 2000.

[14] Prosky L Asp NG Schweizer TF Devries JW Furda I Lee SC. Determination of soluble dietary fiber in foods and food products: Collaborative study. J AOAC Int. 1994;77(3):690–4. 10.1093/jaoac/77.3.690 8012222

[15] Merrill AL, Watt BK, editors. Energy value of foods: Basis and derivation. Agriculture handbook no. 74. Washington DC, USA: U.S. Government Printing office; 1973.

[16] Tavares RL Silva AS Campos ARN Schuler ARP Aquino JS. Nutritional composition, phytochemicals and microbiological quality of the legume, *Mucuna pruriens.* Afr J Biotechnol. 2015;14(8):676–82. 10.5897/AJB2014.14354

[17] Santos PDS Silveira R Reis NV Visentainer JV Santos OO. Analytical method development for fatty acid direct methylation in fruits. J Braz Chem Soc. 2018;29(8):1645–52. 10.21577/0103-5053.20180036

[18] Libraries WS. Wiley Registry® of Mass Spectral Data. Aarle-Rixtel, the Netherlands: John Wiley & Sons; 2020. Available from: https://www.mswil.com/software/spectral-libraries-and-databases/wiley-spectral-libraries/.

[19] Rigoli IC Schmitt CC Ramos LA Neumann MG Cavalheiro ETG. Thermal behaviour of TEGMMA copolymers obtained by photopolymerization using iron complexes. Eclét Quím. 2007;32(3):39–44. 10.1590/S0100-46702007000300006

[20] Cheary RW Coelho AA Cline JP. Fundamental parameters line profile fitting in laboratory diffractometers. J Res Natl Inst Stand Technol. 2004;109(1):1–25. 10.6028/jres.109.002 27366594 PMC4849620

[21] Rheo3000, v. 2.2.28 software, Brookfield AMETEK, Stoughton, WI, USA; 2011. Available from: http://rheo3000.blogspot.com/search/label/Patch.

[22] Holdsworth SD. Applicability of rheological models to the interpretation of flow and processing behavior of fluid food products. J Texture Stud. 1971;2(4):393–418. 10.1111/j.1745-4603.1971.tb00589.x 28370126

[23] Duffy JJ Rega CA Jack R Amin S. An algebraic approach for determining viscoelastic moduli from creep compliance through application of the generalised Stokes-Einstein relation and Burgers model. Appl Rheol. 2016;26(1):15130. 10.3933/applrheol-26-15130

[24] IBM SPSS Statistics for Windows, v. 24.0, trial version, IBM Corp., Armonk, NY, USA; 2016. Available from: https://www.ibm.com/br-pt/analytics/spss-trials.

[25] Tapia MS, Alzamora SM, Chirife J. Effects of water activity (*a*_w_) on microbial stability: As a hurdle in food preservation. In: Barbosa‐Cánovas GV, Fontana Jr AJ, Schmidt SJ, Labuza TP, editors. Water activity in foods: Fundamentals and applications. Hoboken, NJ, USA: Wiley- Blackwell; 2007. 10.1002/9780470376454.ch10 10.1002/9780470376454.ch10

[26] Filip M Vlassa M Coman V Halmagyi A. Simultaneous determination of glucose, fructose, sucrose and sorbitol in the leaf and fruit peel of different apple cultivars by the HPLC-RI optimized method. Food Chem. 2016;199:653–9. 10.1016/j.foodchem.2015.12.060 26776021

[27] Elleuch M Bedigian D Roiseux O Besbes S Blecker C Attia H. Dietary fibre and fibre-rich by-products of food processing: Characterisation, technological functionality and commercial applications: A review. Food Chem. 2011;124(2):411–21. 10.1016/j.foodchem.2010.06.077

[28] Martínez R Torres P Meneses MA Figueroa JG Pérez-Álvarez JA Viuda-Martos M. Chemical, technological and *in vitro* antioxidant properties of mango, guava, pineapple and passion fruit dietary fibre concentrate. Food Chem. 2012;135(3):1520–6. 10.1016/j.foodchem.2012.05.057 22953888

[29] Mudgil D Barak S. Composition, properties and health benefits of indigestible carbohydrate polymers as dietary fiber: A review. Int J Biol Macromol. 2013;61:1–6. 10.1016/j.ijbiomac.2013.06.044 23831534

[30] Stallings VA, Harrison M, Oria M, editors. Dietary reference intakes for sodium and potassium. Washington, DC, USA: The National Academies Press; 2019. 10.17226/25353 10.17226/25353 30844154

[31] Fujita E Vieites RL Daiuto ER Smith RE. Refrigerated storage of the fruits of buriti (*Mauritia flexuosa* L.). Adv Hortic Sci. 2014;28(1):3–8. 10.13128/ahs-22745

[32] Marques LG Prado MM Freire JT. Rehydration characteristics of freeze-dried tropical fruits. Food Sci Technol. 2009;42(7):1232–7. 10.1016/j.lwt.2009.02.012

[33] Berto A Silva AF Visentainer JV Matsushita M Souza NE. Proximate compositions, mineral contents and fatty acid compositions of native Amazonian fruits. Food Res Int. 2015;77(3):441–9. 10.1016/j.foodres.2015.08.018

[34] Institute of Medicine. Dietary reference intakes for vitamin A, vitamin K, arsenic, boron, chromium, copper, iodine, iron, manganese, molybdenum, nickel, silicon, vanadium, and zinc. Washington, DC, USA: The National Academies Press; 2001. 10.17226/10026 10.17226/10026 25057538

[35] Berry SE Mulla UZ Chowienczyk PJ Sanders TAB. Increased potassium intake from fruit and vegetables or supplements does not lower blood pressure or improve vascular function in UK men and women with early hypertension: A randomised controlled trial. Br J Nutr. 2010;104(12):1839–47. 10.1017/S0007114510002904 20673378

[36] Konczak I Roulle P. Nutritional properties of commercially grown native Australian fruits: Lipophilic antioxidants and minerals. Food Res Int. 2011;44(7):2339–44. 10.1016/j.foodres.2011.02.023

[37] Tian T Zhao Y Hunag Q Li J. n-3 polyunsaturated fatty acids improve inflammation *via* inhibiting sphingosine kinase 1 in a rat model of parenteral nutrition and CLP-induced sepsis. Lipids. 2016;51(3):271–8. 10.1007/s11745-016-4129-x 26856322

[38] Lee H Park WJ. Unsaturated fatty acids, desaturases, and human health. J Med Food. 2014;17(2):189–97. 10.1089/jmf.2013.2917 24460221

[39] Amorim VR Rodrigues DCN Silva JN Ramos CLS Almeida LMN Almeida AAC Anti-inflammatory mechanisms of fruits and by-products from *Mauritia flexuosa*, an exotic plant with functional benefits. J Toxicol Environ Health A. 2021;84(11):441–57. 10.1080/15287394.2021.1881672 33641623

[40] Marangoni F Poli A. Phytosterols and cardiovascular health. Pharmacol Res. 2010;61(3):193–9. 10.1016/j.phrs.2010.01.001 20067836

[41] Corrêa SC Clerici MTPS Garcia JS Ferreira EB Eberlin MN Azevedo L. Evaluation of dehydrated marolo (*Annona crassiflora*) flour and carpels by freeze-drying and convective hot-air drying. Food Res Int. 2011;44(7):2385–90. 10.1016/j.foodres.2011.02.052

[42] Gill P Moghadam TT Ranjbar B. Differential scanning calorimetry techniques: Applications in biology and nanoscience. J Biomol Tech. 2010;21(4):167–93. 21119929 PMC2977967

[43] Bernal C Couto AB Breviglieri ST Cavalheiro ETG. Influence of some experimental parameters on the results of differential scanning calorimetry - DSC. Quim Nova. 2002;25(5):849–55. 10.1590/S0100-40422002000500023

[44] Caparino OA Tang J Nindo CI Sablani SS Powers JR Fellman JK. Effect of drying methods on the physical properties and microstructures of mango (Philippine ‘Carabao’ var.) powder. J Food Eng. 2012;111(1):135–48. 10.1016/j.jfoodeng.2012.01.010

[45] Yu S Ma Y Menager L Sun DW. Physicochemical properties of starch and flour from different rice cultivars. Food Bioprocess Technol. 2010;5(2):626–37. 10.1007/s11947-010-0330-8

[46] Mosquera LH Moraga G Martínez-Navarrete N. Effect of maltodextrin on the stability of freeze-dried borojó (*Borojoa patinoi* Cuatrec.) powder. J Food Eng. 2010;97(1):72–8. 10.1016/j.jfoodeng.2009.09.017

[47] Carneiro LR, editor. Production of acerola powder: Drying methods and stability evaluation [MSc Thesis]. Fortaleza, Brazil: Universidade Federal do Ceará; 2014.

[48] Wendin K Ekman S Margareta B Olle E Daniel J Elisabet R Objective and quantitative definitions of modified food textures based on sensory and rheological methodology. Food Nutr Res. 2010;54:5134. 10.3402/fnr.v54i0.5134 20592965 PMC2894641

[49] Berski W Ptaszek A Ptaszek P Ziobro R Kowalski G Grzesik M Pasting and rheological properties of oat starch and its derivatives. Carbohydr Polym. 2011;83(2):665–71. 10.1016/j.carbpol.2010.08.036

